# Piecing together the puzzle: current knowledge and open questions about the *Klebsiella pneumoniae* type VI secretion system (T6SS)

**DOI:** 10.1590/0074-02760260010

**Published:** 2026-07-17

**Authors:** Leticia Miranda Santos Lery, Paulo Ricardo Batista

**Affiliations:** 1 Fundação Oswaldo Cruz-Fiocruz, Instituto Oswaldo Cruz, Laboratório de Microbiologia Celular, Rio de Janeiro, RJ, Brasil; 2 Fundação Oswaldo Cruz-Fiocruz, Programa de Computação Científica, Rio de Janeiro, RJ, Brasil

**Keywords:** VgrG, phospholipase, effector proteins, bacterial virulence, gene expression regulation

## Abstract

*Klebsiella pneumoniae* (Kp) is currently a top priority for the development of alternative therapeutic strategies, according to the World Health Organisation, due to serious concerns regarding infections caused by multiple drug resistant (MDR) bacteria. The convergence of hypervirulence and MDR represents a major threat to public health worldwide. Kp displays an extensive genomic diversity, reflecting a variable repertoire of resistance and virulence factors. Despite this heterogeneity, the type VI secretion system (T6SS) is encoded in most Kp-genomes and plays important roles in competition and pathogenesis. The T6SS is a large macromolecular complex assembled in the cytoplasm that spans the inner and outer membranes. Upon activation, it undergoes conformational changes allowing the delivery of effectors into the extracellular milieu or target cells. Herein, we summarise the current knowledge on the Kp-T6SS, presenting a chronological overview of published studies, discussing the mechanisms, signals, and regulators involved in T6SS expression. Next, we detail both predicted and characterised effector proteins, including Tle1, Pld1 and VgrG4. Finally, we discuss the presence of Kp-T6SS in mobile genetic elements or in clinical samples, highlighting the importance of genomic vigilance and Kp-T6SS detection in high-risk cases. This review integrates available evidence, identifies knowledge gaps, and outlines future directions.

## The T6SS puzzle

The type VI secretion system (T6SS) is a sophisticated and dynamic nanomachine that enables Gram-negative bacteria to translocate effector molecules either into the extracellular milieu or directly into target cells. A typical T6SS is a macromolecular complex assembled within the bacterial cytoplasm, anchored to the inner membrane (membrane complex: proteins TssJ, TssL and TssM; and baseplate: TssE TssF, TssG, TssK), and composed of a contractile sheath (VipA/VipB proteins or TssB/TssC) encasing an inner tube (Hcp or TssD proteins) that can traverse both the inner and outer bacterial membranes upon contraction. At the top of the tube, there is a tip (VgrG and PAAR proteins); the tube and tip components may associate with effector proteins or contain effector domains (Table I). The contraction-driven mechanism propels the tube, tip, and associated effectors toward target cells, allowing their delivery, thus enabling its functional impact.^1^ ^,2^ Beyond its established role in bacterial competition, the T6SS has also been implicated in cooperative behaviours, interactions with other microorganisms and eukaryotic hosts, and nutrient acquisition.^3^ ^,4^ ^,5^ ^,6^ ^,7^

**TABLE I:** T6SS components grouped according to their structural and functional roles

Standardised gene nomenclature	Protein homologs	Structural and functional roles
*tssJ*	VasD/Lip/SciN	Membrane complex
*tssL*	IcmH/DotU/VasF	
*tssM*	IcmF/VasK	
*tssE*	HsiF	Baseplate
*tssF*	VasA/ImpG	
*tssG*	VasB/ImpH	
*tssK*	VasE/ImpJ	
*tssB*	VipA/ImpB	Outer contractile sheath
*tssC*	VipB/ImpC	
*tssH*	ClpV/VasG	AAA+ ATPase, sheath circulation
*tssD*	Hcp	Inner tube, effector
*tssA*	VasJ/ImpA	Tube cap
*tagA*	TagA	Tube stopper
*tssI*	VgrG	Tip, effector
*tagD*	PAAR	Tip

Standardised *tss* (type VI secretion system) gene nomenclature and commonly used homologous protein names are shown. Adapted from Liu et. al.^53^

Moreover, T6SS is envisaged as an engineerable protein delivery nanomachine, with possible biotechnological applications.^8^ ^,9^ For example, Hersch et al. engineered the *Vibrio cholerae* T6SS to deliver Cre recombinase into target cells, demonstrating its potential for genetic editing; and used the system to deliver the effector TseC to kill *Pseudomonas aeruginosa*, highlighting its potential for targeted microbial control.^8^ More recently, Pérez-Lorente et al. engineered the *Pseudomonas putida* T6SS to heterologously deliver diverse effectors, including Tse1 from *Pseudomonas chlororaphis*, which induced sporulation in plant-beneficial Bacillus strains, and TplE from *P. aeruginosa*, which inhibited *Aeromonas hydrophila*.^9^ Antifungal activity was also achieved through delivery of Tfe2 from *Serratia marcescens*, causing cellular damage in *Botrytis cinerea*. Notably, the system was also able to deliver non-T6SS proteins such as chitosanase, demonstrating its versatility. These studies illustrate the potential of engineered T6SS platforms for targeted antimicrobial activity and the development of biocontrol strategies in agriculture.

The structure of the T6SS has been progressively unravelled in the past two decades, mainly due to increasing capacity of advanced techniques for elucidation of protein-protein interactions in large macromolecular complexes.^3^ ^,4^ ^,10^ ^,11^ ^,12^ As a result, the molecular mechanisms underlying T6SS function are now considerably better understood. It is well established that tube and tip components (VgrG/PAAR/Hcp proteins) may carry extended functional domains with effector roles, referred to as "evolved effectors". An additional class of effectors requires specific interactions with cognate VgrG, PAAR, or Hcp proteins to be loaded on T6SS apparatus. To date, hundreds of effectors have been described. They have also been shown to contribute to the stability and assembly of the secretion system itself.^13^ Proper effector loading frequently requires dedicated chaperones or adaptor proteins. These aspects of T6SS architecture and function have been comprehensively reviewed elsewhere^14^ and therefore are not discussed in detail in the present review.

Although the designation "T6SS" was formally proposed in 2006, following its initial descriptions in *V. cholerae* and *P. aeruginosa*,^1^ ^,2^ earlier studies had already identified homologous components in other bacteria, such as *Rhizobium* and *Edwardsiella* species.^15^ ^,16^ These findings underscore that elements of this complex were recognised as virulence-associated factors prior to its comprehensive characterisation. Most of the current knowledge of T6SS biology derives from studies in *Escherichia coli*, *P. aeruginosa* and *V. cholerae*. While aspects of T6SS structure, function, and regulation have been investigated in other species, the available data remain relatively sparse. In *Klebsiella pneumoniae*, significant progress has been made toward characterising the T6SS; nevertheless, substantial gaps in our understanding persist. In the following sections, we summarise the available knowledge and highlight key questions that remain to be addressed.

## The timeline of *K. pneumoniae* T6SS pieces

**Fig. 1:**
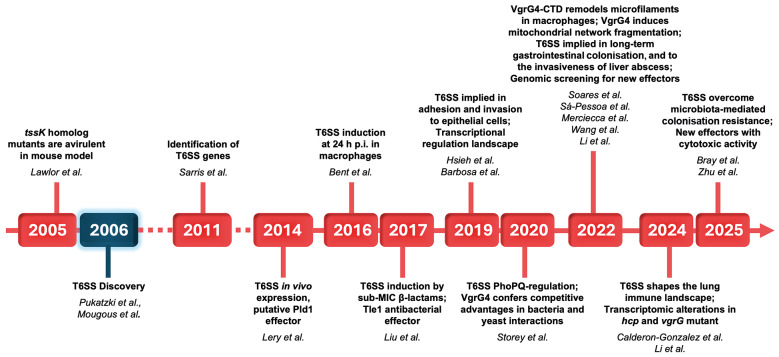
timeline of key discoveries associated with the functional characterisation of the *Klebsiella pneumoniae* T6SS. Major findings are shown in black text, with key references cited in *italic*. The corresponding publication years are indicated along the red timeline, while the blue box highlights the year of the initial description of T6SS in the literature. p.i.: post-infection.

In this section, we chronologically summarise the main studies and key findings related to the T6SS in *K. pneumoniae* (Fig. 1). Herein we describe studies elucidating the role of T6SS in interbacterial competition, host colonisation, and pathogenesis, as well as studies addressing T6SS effectors and regulatory mechanisms.

An early study by Lawlor et al. in 2005 provided functional evidence for previously unrecognised virulence-associated *loci* in *K. pneumoniae*.^17^ Using a large-scale screening of approximately 4,800 transposon insertion mutants in a mouse model, after infection, the authors identified 106 independent mutants that failed to be recovered from the lungs or spleens of infected mice.

Notably, several of these mutants harboured insertions in hypothetical open reading frames (ORFs), suggesting that previously uncharacterised factors may contribute to bacterial pathogenesis. Two of the mutants displaying reduced ability to colonise the mouse spleen had insertions in hypothetical ORFs, showing significant sequence similarity to the *Yersinia pestis* protein YP01467. This protein was later annotated as TssK, a core component of the T6SS baseplate. Although this gene was not recognised as part of the T6SS at the time of the study, these findings represent the first evidence implicating a *K. pneumoniae* T6SS component in virulence.

A few years later, in 2011, the first report of the T6SS in *Klebsiella* was made by Sarris et al.^18^ Considering the growing number of studies about T6SS relevance for bacterial competition and virulence in other species, the authors investigated the presence of genes encoding putative T6SS core components and effectors in *Klebsiella* spp. genomes. Sequence similarity analysis was performed in three fully sequenced *K. pneumoniae* strains (*K. pneumoniae* 342, *K. pneumoniae* NTUH-K2044, and *K. pneumoniae* subsp. *pneumoniae* MGH 78578), one partially sequenced strain (*K. pneumoniae* subsp. *rhinoscleromatis* ATCC 13884), and the *Klebsiella variicola* strain At22. *In silico* analysis revealed the presence of T6SS genes in these genomes, organised into up to three conserved syntenic *loci* per genome.

In 2014, Lery et al. sequenced additional *K. pneumoniae* genomes and published a comparative genomics study.^19^ T6SS genes were detected in the genomes of most strains. Moreover, T6SS mRNA expression was detected when the Kp52.145 strain colonised mouse lungs, suggesting that those genes might be induced by host-associated stimuli. The authors also reported that some T6SS *loci* contained strain-specific insertions encoding putative T6SS effectors. Notably, in the *K. pneumoniae* 52.145 strain, a K2 hypervirulent strain, one T6SS *locus* contained an insertion encoding putative phospholipase D (*pld*) genes. A *pld1*-deficient mutant was avirulent in a mouse pneumonia model, providing the first evidence for a putative *K. pneumoniae* T6SS effector directly implicated in virulence.

In 2016, Bent et al. developed a method for enrichment of pathogen transcripts from eukaryotic host cells, which they applied to analyse the interaction between *K. pneumoniae* ATCC BAA-2146 and murine macrophages P388D1.^20^ Using this approach, the authors observed increased expression of T6SS-associated genes after 24 h of infection. These findings are consistent with previous studies, indicating that the T6SS is not constitutively expressed in *K. pneumoniae*. Moreover, these data reinforce that *K. pneumoniae* T6SS might have a role in the modulation of the host response to infection.

In a subsequent study, Liu et al. identified the Tle1 (Type VI lipase effector) in the MDR *K. pneumoniae* strain HS11286.^21^ This study showed that Tle1 was responsible for antibacterial activity against *E. coli.* Of note, Tle1 shares no significant sequence similarity with the putative phospholipase Pld1, highlighting the diversity of T6SS-associated effectors. Additionally, this study showed that sub-inhibitory concentrations of β-lactam antimicrobials induced the T6SS activity.

A broader view of T6SS regulation was provided by Barbosa et al. in 2019,^22^ suggesting that *K. pneumoniae* T6SS expression might be regulated in response to specific environmental signals sensed within the human host. Through the prediction of transcriptional regulator binding sites upstream of T6SS gene transcriptional start sites, the study associated T6SS regulation with temperature (H-NS), nutrient limitation (GcvA and Fis), oxidative stress (OxyR), and osmolarity (RscAB and OmpR).

Since 2019, the number of studies investigating the *K. pneumoniae* T6SS has increased substantially. Hsieh et al. reported that about 88% of *K. pneumoniae* isolates causing pyogenic liver abscesses (PLA) were T6SS-positive, while only 41% were T6SS-positive among the *K. pneumoniae* isolates from intestinal colonisation.^23^ The authors further demonstrated that the PLA-derived strain NTUH-K2044 mutated in genes *icmF1/icmF2* (*tssM*) was attenuated *in vivo* and presented reduced adhesion and invasion to intestinal cells. In addition, they showed that the histone-like nucleoid structuring protein (H-NS) binds the regulatory region and represses expression of *tssD* (hcp, the tube component).

In 2020, Storey et al. presented a comprehensive analysis of T6SS in the Kp52.145 strain.^24^ They found that T6SS is positively regulated by the PhoPQ two-component system, as well as PmrAB, Hfq, Fur, RpoS and RpoN; whereas H-NS act as a negative regulator. In addition, they showed that the VgrG4 protein confers a competitive advantage to *K. pneumoniae* during interaction with bacterium and yeast. Such effects were attributed to its domain of unknown function, DUF2345. VgrG4 exerts antibacterial activity via reactive oxygen species (ROS) generation, whereas the antitoxin Sel1E protects *K. pneumoniae* from VgrG4-mediated toxicity. A few years later, Sá-Pessoa et al. demonstrated that VgrG4 triggers the fragmentation of the mitochondrial network in *Saccharomyces cerevisiae*.^25^ Interestingly, in 2022, Soares et al. described that the C-terminal region of the VgrG4 protein induces remodelling of actin filaments in macrophages.^26^ The consequences of such modulation for bacterial infection remain to be elucidated, although it is increasingly clear that VgrG4 has multiple roles in the interaction of *K. pneumoniae* with both bacterial and eukaryotic cells.

To further explore possible effectors, Li et al. performed an in-depth comparative genomic analysis of the T6SS in 241 sequenced strains of *K. pneumoniae*.^27^ The authors assessed the synteny of the T6SS *loci* in different isolates, as well as predicted effectors. The T6SS was frequently found in *K. pneumoniae* genomes, and the presence of two *loci* encoding T6SS genes was the most prevalent gene organisation. Moreover, the authors found that a variable region downstream of a *vgrG* gene usually encodes effector proteins. Conserved domain analysis indicated that the putative effectors may have roles such as lipases, ribonucleases, deoxyribonucleases, and polysaccharide hydrolases.

Merciecca et al. in 2022 found that T6SS-1 isogenic mutants colonised the gastrointestinal tract of mice less efficiently than the wild-type strain over the long term.^28^ Comparative analysis of faecal 16S rRNA sequences indicated that T6SS-1 reduced the microbiota richness and its resilience capacity. Oscillospiraceae family members were identified as specific competitors for the long-term gut establishment of *K. pneumoniae*. To investigate the underlying competition molecular mechanisms, the authors heterologously expressed the *K. pneumoniae* Tle1 effector in *E. coli* periplasm. As result, the bacterial cell membrane permeability was affected, revealing a possible mechanism of action. In addition, the authors identified a gene specifically encoded in *Klebsiella* species whose product was predicted to be a putative effector, designated Tke (type VI *Klebsiella* effector). Its role remains to be determined.

Also in 2022, Zhang et al. investigated the role of the stationary-phase transcriptional regulator BolA in *K. pneumoniae*.^29^ By comparing wild-type and *bolA* mutant strains using quantitative real-time polymerase chain reaction (qRT-PCR), they found that the T6SS genes *vgrG* and *clpV* were transcriptionally regulated by BolA. Using a similar approach, Fan et al., compared wild-type and integration host factor (*ihf*) mutant using RNAseq, revealing that some T6SS genes, including *tssG* and *hcp*, were also modulated by this regulator.^30^ Intriguingly, transcript sequencing and quantitation by Zhou et al., in 2023, comparing wild-type and *rpoS* mutant of an ESBL-producing hypervirulent isolate, did not identify modulation of T6SS genes, contrasting with previous findings of Storey et al.^31^ These observations suggest that regulatory mechanisms may vary in different strains and growth conditions.

In 2024, Li et al. investigated the effects of knocking out the T6SS marker genes *hcp* or *vgrG*.^32^ In both mutants, expression of other T6SS genes within *locus* I was undetected. Transcriptomic analysis revealed that the majority of genes modulated in the *hcp* mutant compared to the wild type were similarly modulated in the *vgrG* mutant. Notably, a significant proportion of bacterial genes were modulated in the absence of *hcp* or *vgrG*, with 1,298 genes upregulated and 1,752 downregulated in both mutants. Additionally, interbacterial competition experiments showed that both *hcp* and *vgrG* were essential for the competitive ability of the ST11 *K. pneumoniae* HS11286.

That same year, Calderón-González et al. contributed to a detailed understanding of the molecular mechanisms of pathogenesis by demonstrating that the T6SS shapes the pulmonary immune landscape. Specifically, T6SS activity modulated bacterial interactions with monocytes and macrophages by promoting a shift from alveolar to interstitial macrophages and limiting infection of inflammatory monocytes.^33^ The absence of T6SS increased the number of cells expressing markers of active cells and decreased the subpopulations expressing the immune checkpoint PD-L1. Therefore, the T6SS might have a role in aiding bacterial infection persistence.

More recently, Bray et al. showed that T6SS genes are directly regulated by ArgR, FNR, and Fur in response to gut-specific growth conditions, reinforcing the notion that *K. pneumoniae* employs the T6SS to overcome microbiota-mediated colonisation resistance by reducing the Betaproteobacteria members in a T6SS-dependent manner.^34^ In line with these findings, Zhao et al., in 2025, proposed the T6SS as a predictor of subsequent bloodstream infection (sBSI) in patients carrying carbapenem-resistant (CR-KP) strains on intestinal colonisation.^35^ Such patients were more frequently associated with prior invasive procedures, antibiotic exposure, and immunosuppression, and showed a strong association with 28-day mortality. Moreover, T6SS-positive CR-KP strains exhibited a higher prevalence of virulence genes, such as *rmpA* and *iucA*, compared to T6SS-negative isolates. Notably, strains from the sBSI group displayed significantly increased *hcp* and *vgrG* mRNA expression relative to colonisation isolates, suggesting that key T6SS components may contribute to the occurrence and progression of CR-KP–associated sBSI.

Finally, Zhu et al. employed statistical and computational approaches to predict novel T6SS effector proteins.^36^ The authors experimentally validated the antagonistic activity against *E. coli* for effectors harbouring DUF3258, DUF3751, and Sel1 domains.

Collectively, the studies described above illustrate the significant progress made in recent years toward understanding the *K. pneumoniae* T6SS. Substantial information is now available regarding transcriptional regulators of T6SS genes, as well as environmental signals that trigger T6SS expression or activity. Several effector proteins have also been characterised, revealing roles in bacterial competition, colonisation, modulation of cell-cell interactions, and pathogenesis. In addition, multiple studies have examined the prevalence of T6SS-positive isolates in different sample groups and the genomic contexts of T6SS loci. Further details on these aspects are provided below. Throughout the following sections, we highlight remaining knowledge gaps that must be addressed to fully assemble the T6SS puzzle.

## T6SS expression and regulatory mechanisms in *K. pneumoniae*

The expression of the T6SS in *K. pneumoniae* is not constitutive. Reports show that T6SS gene expression is increased when bacteria colonise the lungs of a mouse pneumonia model, as well as after 24 h of *in vitro* interaction with murine macrophages.^19^ ^,20^ Additionally, sub-inhibitory concentrations of β-lactams were reported to induce T6SS expression.^21^ The two-component systems (TCSs) PhoPQ and PmrAB were shown to positively regulate T6SS expression in the Kp52.145 strain.^24^ Both systems are associated with antimicrobial resistance, more specifically with resistance to polymyxins, induced through the modifications of lipid A and bacterial surface charge. Altogether, these data point to the relevance of T6SS in the context of pathogenesis and to a regulatory crosstalk between virulence and resistance mechanisms (Table II). The correlation between T6SS and antimicrobial resistance is further discussed in the section "T6SS genomic context and distribution".

**TABLE II:** Summary of studies identifying conditions and regulators involved in T6SS expression in *Klebsiella pneumoniae*

Authors	Year	Regulatory mechanism / regulator	Strain(s)
Lery et al.	2014	T6SS gene expression detected during lung infection in a murine pneumonia model	Kp52.145
Bent et al.	2016	Increased T6SS gene expression 24 h p.i. in murine macrophages (P388D1)	ATCC BAA-2146
Liu et al.	2017	T6SS activity induced by sub-inhibitory concentrations of β-lactam antimicrobials	HS11286
Barbosa et al.	2019	Predicted transcription factor binding sites upstream of T6SS genes, including H-NS (temperature), GcvA and Fis (nutrient limitation), OxyR (oxidative stress), and RcsAB and OmpR (osmolarity)	Kp52.145, HS11286, NTUH-K2044
Hsieh et al.	2019	Histone-like nucleoid structuring protein (H-NS) binds regulatory region and represses *tssD* (*hcp*) expression	NTUH-K2044
Storey et al.	2020	Positive regulation by PhoPQ two-component system; PmrAB, Hfq, Fur, RpoS, and RpoN act as additional positive regulators; H-NS acts as a negative regulator	Kp52.145
Zhang et al.	2022	*vgrG* and *clpV* transcriptionally regulated by BolA	NTUH-K2044
Fan et al.	2023	*tssG* and *hcp* modulated by integration host factor (Ihf)	W14
Bray et al.	2025	*tssB* and *tssK2* directly regulated by ArgR, Fnr, and Fur under gut-associated growth conditions	KPPR1S

p.i.: post-infection.

There is also a co-regulation of T6SS with other virulence mechanisms in *K. pneumoniae.* For instance, the ferric uptake regulator (Fur), which controls iron acquisition and the expression of siderophores, capsule, fimbriae and LPS, was shown to positively regulate T6SS in Kp52.145 and KPPR1S strains.^24^ ^,34^ Similarly, the integration host factor (*Ihf*), a global regulator of multiple virulence determinants, positively modulates T6SS genes expression in the W14 strain.^30^

Moreover, conserved sequences with the binding boxes of the following regulators were identified upstream of the transcriptional start sites of T6SS genes of at least three frequently studied strains: H-NS, Fis, GcvA, OxyR, RscAB and OmpR.^22^ Interestingly, H-NS was confirmed as a negative regulator in NTUH-K2044 and Kp52.145 strains.^23^ ^,24^ As a pleiotropic regulator, H-NS has been shown to modulate virulent traits in *K. pneumoniae*, including the downregulation of capsule.

T6SS gene expression is also associated with starvation and stress conditions. In the NTUH-K2044 strain, T6SS genes were positively regulated by BolA,^29^ a conserved transcriptional regulator in Gram-negative bacteria, involved in flagella biosynthesis, biofilm formation, stress responses, and iron metabolism, and has also been implicated in antibiotic response and bacterial virulence. In the Kp52.145 strain, the general stress response sigma factor RpoS, and the sigma factor RpoN are positive regulators of T6SS genes. Curiously, in HKE9 strain, RpoS does not modulate T6SS gene expression.^31^ Transcriptomic analysis by Zhou et al., comparing wild-type and the *rpoS* mutant of an ESBL-producing hypervirulent isolate did not find T6SS genes modulated, contrasting to previous findings of Storey et al.^24^ Thus, the genomic diversity observed among *K. pneumoniae* isolates is also reflected in differences between T6SS regulatory mechanisms, reinforcing the importance of studying molecular mechanisms in multiple strains.

**Fig. 2:**
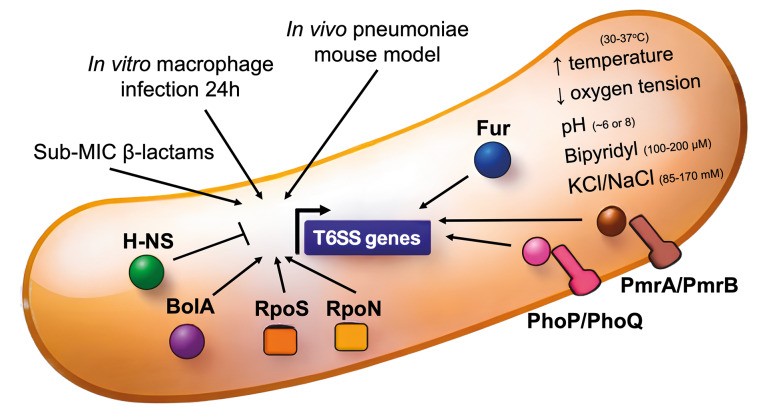
regulators, regulatory mechanisms and signals described as associated with T6SS expression/activity. Arrows indicate induction or activation conditions, while the T-bar indicates inhibition. Sub-MIC: under the minimum inhibitory concentration.

With several putative regulators identified, some experimentally confirmed, a framework for T6SS regulation has been proposed (Fig. 2). However, important gaps remain regarding the temporal and fine-tuning regulatory mechanisms, the existence of post-transcriptional regulatory mechanisms and the differences between strains.

## *Klebsiella pneumoniae* T6SS effectors

Effectors are the molecules translocated through secretion systems. Because they are delivered into target cells and directly interact with host or competitor cell components, they are essential mediators of secretion system activity. To date, there are several *K. pneumoniae* T6SS effectors predicted, but very few have already been characterised (Table III).

**TABLE III:** Studies that contributed to the knowledge of T6SS effectors in *Klebsiella pneumoniae*

Authors	Year	Effector	Phenotype / function	Strain
Lery et al.	2014	Pld1 - putative phospholipase D1	avirulent in mouse model	Kp52.145
Liu et al.	2017	Tle1 - T6SS lipase effector	antibacterial activity against *Escherichia coli*	HS11286
Storey et al.	2020	VgrG4 (DUF2345)	promotes competitive advantages during interaction with bacteria and yeasts	Kp52.145
Soares et al.	2022	VgrG4 (C-terminal extension)	induces remodelling of actin filaments in macrophages	Kp52.145
Li et al.	2022	Tde, Tle, Tse, Tpe, others	computational prediction and domain analysis	241 strains
Merciecca et al.	2022	Tle1, Tke	T6SS-1 plays a role in long-term gastrointestinal colonisation	CH1157
Sá-Pessoa et al.	2023	VgrG4 (DUF2345)	triggers the fragmentation of the mitochondrial network in *Saccharomyces cerevisiae*	Kp52.145
Zhu et al.	2025	proteins harbouring DUF3258, DUF3751, and Sel1 domains	antibacterial activity against *E. coli*	multiple strains

**Fig. 3:**
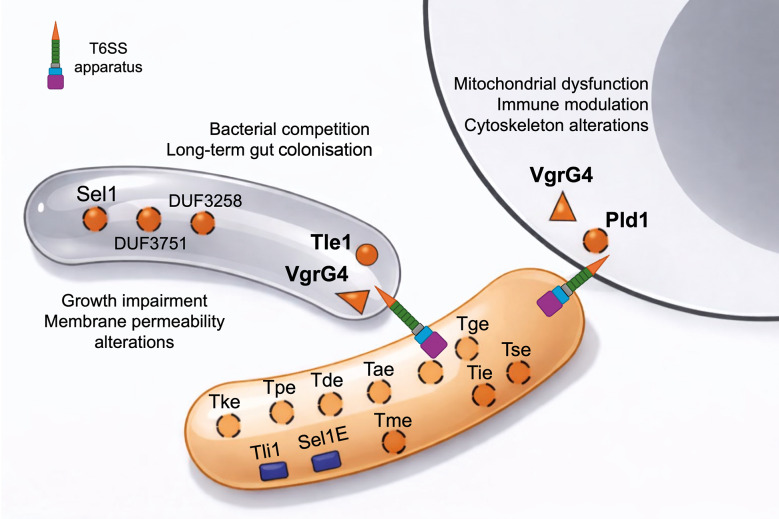
effectors associated with the *Klebsiella pneumoniae* T6SS. Both antibacterial and anti-eukaryotic effectors proposed to date are depicted. Effectors with experimentally demonstrated T6SS-dependent activity, including VgrG4 and Tle1, are represented with solid black outlines. Their cognate immunity proteins, Sel1E and Tli1, are shown as blue rectangles. Effectors identified through computational prediction or exhibiting partial functional characterisation are indicated with dashed outlines.

With respect to antibacterial activity, the Type VI lipase effector (Tle1) is the most studied so far (Fig. 3). Its role has been initially demonstrated in the reference strain HS11286, highlighting Tle1 contribution to bacterial competition against *E. coli*.^21^ A homologue in the CH1157 strain was shown to play a role in long-term gastrointestinal colonisation.^28^ The heterologous expression of *K. pneumoniae* Tle1 in the *E. coli* periplasm induces growth retardation and alterations in cell permeability, suggesting it might hydrolyse phospholipids in target cells, functionally similar to Tle from other species.^21^ ^,28^ Tli1 was identified as the cognate immunity protein, neutralising Tle1.^21^ Despite these findings, detailed molecular mechanisms of Tle1 action, phospholipid preferences and binding modes are yet to be deciphered.

Additional effectors with putative antibacterial activity were recently proposed by Zhu et al.^36^ (Fig. 3). The authors analysed nearly 4500 genomes from the *Klebsiella* genus and found 50 effector protein groups. The structural domains encoded in putative effector proteins included 8 main functional categories: peptidase effector (Tpe), DNase and RNase effector (Tde), lipase effector (Tle), amidase effector (Tae), membrane-disrupting effector (Tme), metal iron acquisition effector (Tie), glycoside hydrolase effector (Tge) and unclassified secretion system effector (Tse). Among these candidates, the authors selected 3 representative proteins containing domains not previously described as T6SS effectors, for experimental validation. The genes KP117_01723, KP186_04383, and KP122_03665 coding for proteins containing DUF3258, DUF3751, and Sel1 domain respectively were cloned and heterologously expressed in *E. coli.* A marked growth reduction was observed in all three cases, indicating that those proteins might be T6SS effectors with an antibacterial action. Many pieces of information were made available through this study, although there are still several questions to be answered concerning structural and mechanistic aspects.

In addition, VgrG4 has been characterised as a T6SS effector protein conferring competitive advantages both against prokaryotic and eukaryotic target cells (Fig. 3). VgrG proteins are structural core components of T6SS and also released into target cells. They may associate with effector proteins or encode effector domains. VgrG4 is an evolved *K. pneumoniae* protein encoding an extension. VgrG4 encodes the domains Phage-GPD and T6-VgrG, and also DUF2345 and an additional C-terminal sequence named CTD. It was demonstrated that the DUF2345 domain is sufficient for the antibacterial and anti-eukaryotic effect,^24^ while the CTD induces remodelling of actin filaments.^26^ Moreover, Sá-Pessoa and colleagues have shown that VgrG4 colocalises with the endoplasmic reticulum (ER) protein mitofusin 2 and promotes Ca²⁺ transfer from the ER to mitochondria.^25^ This Ca²⁺ flux activates the mitochondrial fission regulator Drp1, leading to mitochondrial fragmentation, and stimulates the innate immune receptor NLRX1 to produce ROS, which in turn limit NF-κB activation by modulating degradation of its inhibitor, IκBα. Sel1E is the cognate immunity protein protecting against the toxic activity of VgrG4.^24^

Regarding anti-eukaryotic activity, Pld1 is a putative phospholipase D protein involved in bacterial virulence (Fig. 3). *pld1* is encoded in a T6SS *locus* and predicted as a putative effector by bioinformatic analysis. A transposon mutant interrupting the *pld1* gene is avirulent in a mouse pneumonia model.^19^ Its mechanism of action remains unclear so far, as *in vitro* fat-blot and lipidomic assays did not detect alterations in lipid composition.^37^ The recombinant Pld1 protein has been shown to bind macrophage proteins, including ribosomal, RNA-related, small GTPases, and cytoskeleton-related proteins. These findings suggest that Pld1 may modulate host cell complexes, favouring the infection. Despite of these evidence, Pld1 mechanisms of action and secretion are still unknown. Thus, the avirulent phenotype of the pld1⁻ mutant in a mouse model may result from both direct effects on virulence and indirect effects on bacterial fitness.

## T6SS genomic context and distribution

Genomic diversity in *K. pneumoniae* is huge, with genomes typically ~5-6 Mbp in size and encoding about 5,000-6,000 genes.^38^ Approximately 1,700 genes constitute the core genome, whereas the remainder belong to a highly diverse accessory genome that contributes to a pan-genome likely exceeding 100,000 protein-coding sequences, most of which occur in fewer than 10% of strains. Mobile genetic elements are widespread throughout the genomes.^39^ T6SS *loci* are found in genomic islands with sequence evidence (GC content, flanking integrases, among others) suggesting acquisition via horizontal gene transfer.^19^ T6SS genes are not found in every *K. pneumoniae* genome, although they are present frequently. For instance, Morgado et al. in 2022 analysed the prevalence of T6SS in 390 *Klebsiella* spp. genomes from human, animal, and environmental sources in Brazil.^40^ Among *K. pneumoniae*, isolates, the prevalence of T6SS was 76%. Importantly the genetic *loci* encoding T6SS present variations among strains, including insertion regions possibly coding for additional effectors.^18^ ^,19^ ^,32^ ^,36^

As previously mentioned, the genetic organisation of T6SS in *K. pneumoniae* genomes is also diverse. Recently, Zhu et al. analysed 4,434 genomes and identified two major T6SS *loci*, T6SSkleb1 and T6SSkleb2, which appear to have independent evolutionary origins rather than deriving from a single ancestral duplication event.^36^ These *loci* show notable differences in gene composition: T6SSkleb2 lacks the baseplate component *tssE*, whereas T6SSkleb1 is missing several core genes, including *tssB*, *tssC*, *tssD*, and *tssH*. In both *loci*, *vgrG* genes are located in variable regions associated with diverse effector-immunity modules. Although individual *loci* may lack some T6SS components, most analysed strains carried both T6SS *loci* complete.^36^ Moreover, many genomes harbour "orphan" *hcp* (*tssD*) and *vgrG* (*tssI*) genes outside the main *loci*, often linked to nearby effector genes, further highlighting the modular and dynamic organisation of the *Klebsiella* T6SS.

T6SS is implied in *K. pneumoniae* virulence, colonisation, and pathogenesis. The convergence of hypervirulence (hvKP) and antimicrobial resistance (AMR) in the same strain is a real threat, although it is still described in low frequency.^41^ In 2019, Chen et al. found that T6SS was encoded on a large genomic island of an MDR strain isolated from a pig.^42^ This is interesting to notice that this mobile genomic element encoded both resistance and virulence determinants, revealing the potential for evolution and emergence of risky clones. Moreover, Altayb et al. found four *loci* encoding T6SS genes in an MDR hvKp convergent isolate from Sudan, Africa,^43^ while Takizawa et al. identified T6SS genes in carbapenem resistant strain from a Japan hospital effluent.^44^ Recently, Liu et al. described an hvKP MDR clinical isolate presenting T6SS genes.^45^ Collectively, these reports highlight the importance of genomic monitoring of convergent isolates.

The increase in reports associating *K. pneumoniae* T6SS with virulence led Zhou et al. to analyse the prevalence of T6SS genes among *K. pneumoniae* causing bloodstream infections (BSIs).^46^ Among the clinical isolates analysed, approximately 20% were T6SS-positive. The detection rate of virulence factors, such as *p-rmpA*, *wcaG*, *alls*, *iutA*, *mrkD*, *kfu*, *iucA*, *iroB*, and *entB*, in T6SS-positive strains was significantly higher than in T6SS-negative strains. Thus, the authors concluded that T6SS-positive strains exhibited hypervirulent potential and suggest that clinicians should be aware of the importance of epidemiologic surveillance of T6SS gene clusters.

Further epidemiological studies were performed. For instance, Liao et al. analysed the distribution of T6SS in clinical *K. pneumoniae* strains from a Chinese hospital and T6SS potential relationship with virulence. The T6SS was detected in ~72% of this set of clinical strains, and the T6SS-positive strains presented increased biofilm formation and higher number of virulence genes than T6SS-negative isolates.^47^ Zhang et al. analysed isolates causing bloodstream infections and found that ~16% were T6SS-positive.^48^ The presence of T6SS was significantly correlated with improved competition against *E. coli* and the presence of additional virulence factors. However, in this set of samples, neither an increase in biofilm formation, nor serum resistance was observed in T6SS-positive strains. On the other hand, Wang et al. showed that isolates from invasive liver abscesses (IKPLA) presented increased *hcp* expression, and the T6SS-positive isolates showed higher survival against serum and neutrophil killing.^49^ Moreover, mice infection with T6SS-positive strains had a shorter survival time, higher mortality, and increased interleukin-6 expression in the liver and lungs.

An additional correlational study by Mohamed et al. revealed that among 56 Egyptian samples, the T6SS-positive isolates presented higher resistance rates and biofilm-forming ability.^50^ However, a recent cross-sectional study by Haddadi et al. found that T6SS presence did not significantly correlate with virulence or resistance genes, except for ciprofloxacin resistance.^51^

The studies described in this section provided evidence that T6SS may contribute to serious and life-threatening infections. Therefore, genomic screening of T6SS-positive isolates may be important as a strategy to monitor the risk of infection progression. There is still a lack of development of strategies to inhibit *K. pneumoniae* T6SS expression or activity and assessment if they could contribute to blocking disease or improving bacterial clearance.

## Final remarks: outstanding questions and future directions

Many pieces of the *K. pneumoniae* T6SS puzzle remain missing. Although several transcriptional regulators have been identified, the molecular mechanisms triggering T6SS activation, sheath contraction, tube propulsion, and effector translocation are still poorly understood. For the effectors described so far, their secretion routes remain unknown, including whether they are delivered through association with Hcp, VgrG, PAAR, or other components. Likewise, the existence and roles of additional evolved VgrGs, Hcps, and PAAR proteins remain largely unexplored. Numerous putative effectors have been predicted, but their biological functions await experimental validation. The extensive genomic heterogeneity among *K. pneumoniae* isolates further suggests that additional layers of regulation and functional diversity remain to be uncovered.

Structural biology approaches, particularly cryo-electron microscopy and tomography techniques combined with integrative modelling, have greatly contributed to understanding large macromolecular membrane complexes. So far, they have not been applied to study *K. pneumoniae* T6SS. We highlight these approaches are useful and could improve the knowledge of structure-function relationships on *K. pneumoniae* T6SS.

Finally, it remains to be determined whether a pharmacological inhibition of T6SS expression or activity could prevent or even revert the *K. pneumoniae* infection process. Systematic screening efforts for T6SS inhibitors are still lacking. Although an inhibitor targeting the *E. coli* T6SS was reported in 2021^52^ and proposed to have broader activity due to T6SS conservation, its efficacy against *K. pneumoniae* has not been evaluated. Due to T6SS sequence conservation, the authors suggested that this molecule may possibly inhibit as well *K. pneumoniae* T6SS.

The *K. pneumoniae* is among the pathogens of greatest concern to healthcare systems worldwide. Advancing the understanding of its basic biology, alongside applied research aimed at developing alternative therapeutic strategies for MDR strains, is therefore critical. In this context, the *K. pneumoniae* T6SS represents an emerging field, and contributions from diverse research groups and disciplines will be essential to uncover the missing pieces and integrate them into a more comprehensive picture.

## Data Availability

This article is a review based on previously published studies. No new datasets were generated. All data analysed are available in the cited references.
