# Supplementary material for: Piecing together the puzzle: current knowledge and open questions about the Klebsiella pneumoniae type VI secretion system (T6SS)
Source: Mem Inst Oswaldo Cruz. 2026 Jul 17;121:e260010. doi: 10.1590/0074-02760260010 (PMC13367176; doi:10.1590/0074-02760260010)
Supplement: Peer review history

# Reviewer report: FIRST REVIEW ROUND - REVIEWERS' COMMENTS

## REVIEWER #1

The abstract is properly adequate. The manuscript corresponds to a revision on T6SS in *K. pneumoniae*. The MS is well written and approaches several aspects of the current knowledge and gaps on the proposed subject. The references is appropriate and updated, as well as the presented figures and tables. Find below some comments that I consider relevant for MS improvement.

On page 3, line 36: the authors mentioned the importance of biotechnological application of T6SS. Authors could develop in more detail this issue.

Page 11, lines 26 – 32: In the same way, authors could provide more information on what is known considering the relationship between T6SS activity and resistance, since they provided only one reference on this subject.

Given the complexity of T6SS, it would be interesting to provide a table describing the main function of each T6SS component

Page 16, line 45: this sentence gives the idea that the T6SS would be one of the factors involved with the hypervirulent phenotype in *K. pneumoniae*. Be aware of that since, although T6SS contributes to *K. pneumoniae* pathogenesis, it is not a determinant of hypervirulence. It would be better to remove the reference 46 from this context.

## REVIEWER #2

The manuscript by Lery and Batista is a comprehensive review of the current knowledge regarding the role, regulation, and effectors of the Klebsiella pneumoniae T6SS. Overall, the manuscript is well presented and represents an important contribution to the field. The abstract is adequate, and the figures and tables are valuable additions that greatly help the reader follow the information presented. I have some major points that should be addressed by the authors, as well as some minor revisions to the text, as outlined below.

1) Some *K. pneumoniae* genomes may contain up to three T6SS clusters, including one complete cluster and two incomplete ones. This is an interesting observation that could benefit from further discussion in the manuscript. In particular, the authors do not provide information on the distribution and prevalence of these incomplete clusters. It would be helpful to clarify whether they are conserved across strains or present only in a subset of isolates. In addition, it would be important to specify which clusters are associated with the effectors described in the text. Finally, the authors may wish to comment on whether these incomplete clusters could represent a potential source of variation for the complete cluster.

2) Page 15, lines 39-55 (description of VgrG4 function): The text would benefit from revision to combine sentences that are currently too short. In addition, the mechanism of action of VgrG4 is not clearly explained in the manuscript. It would be helpful for the authors to clarify how VgrG4 induces mitochondrial dysfunction, activates immune receptors, and promotes actin remodeling in a ROS-dependent manner.

3) Page 18, lines 6-8: "Recently, Liu et al. described an hvKP MDR clinical isolate and attributed bacterial hypervirulence due to T6SS – as it lacked hypermucoviscosity": I did not understand the sentence and the correlation of T6SS and hypermucoviscosity. Also, authors mention an association of T6SS presence and biofilm formation but the information it not clearly presented.

- Page 13, lines 3-13: The section describing the regulation of T6SS expression by Fur and IHF would benefit from additional clarification. In particular, it would be helpful to indicate whether these regulators act as positive or negative regulators and under which experimental conditions this regulation was observed. For example, does Fur repress T6SS expression under iron-replete conditions? Same for the text regarding BolA: please describe BolA and how does it regulate T6SS gene expression;

- Authors could also discuss whether some reported effects of lack of T6Ss activity on animal colonization by *K. pneumoniae* could be an indirect effect of reduced competitive fitness against the microbiota or a direct role of the T6SS in virulence (example: page 15, line).

Minor text revisions:

- page 7, line 25 "Notably, in the *K. pneumoniae* 52.145 strain, K2 hypervirulent strain, one T6SS locus": revise to "Notably, in the *K. pneumoniae* 52.145 strain, a K2 hypervirulent strain, one T6SS locus"

-page 11, line 20: "CR-KP strains"- include the full description of the abbreviation.

- Page 14, lines 23-28 (However, there are missing....): sentence needs revision.

- Page 15, line 3: revise to: "With respect to antibacterial activity, the Type VI lipase effector (Tle1) is the best described so far" or "most studied so far"

- page 15, lines 41,42: revise to remove the comma ("the authors selected 3 representative proteins containing domains not previously described as T6SS effectors,")

- Figure 3: my suggestion is to use a T6SS representation instead of a syringe

- Page 17, line 23: "Genomic diversity in *K. pneumoniae* is huge (39).": The information provided would benefit from greater specificity. For example, what is the overall size of the *K. pneumoniae* core genome?

- page 20, lines 18-22: "There is still a lack of systematic screenings efforts for molecules with such activity. Systematic screening efforts for T6SS inhibitors are still lacking.": revise to remove redundancy.

- page 20, line 33: *K. pneumoniae*

## AUTHORS' RESPONSE TO THE REVIEWERS

Dear Editor Ana Carolina Vicente,

We appreciate the time and effort dedicated to the evaluation of our work. We are grateful to you and the reviewers for the constructive and insightful comments provided. We have carefully considered all suggestions and have incorporated the recommended changes in the revised version of the manuscript.

We believe that these revisions have improved the clarity and quality of the work. Below, we provide a detailed, point-by-point response to all reviewer comments and indicate the corresponding modifications made in the manuscript. We hope that the revised manuscript will now be suitable for publication.

Sincerely,

Leticia MS Lery and Paulo Ricardo Batista

Point-by-point reply:

Reviewer: 1:

The abstract is properly adequate. The manuscript corresponds to a revision on T6SS in *K. pneumoniae*. The MS is well written and approaches several aspects of the current knowledge and gaps on the proposed subject. The references are appropriate and updated, as well as the presented figures and tables. Find below some comments that I consider relevant for MS improvement.

Reply: We thank the reviewer for the careful evaluation of our manuscript and are grateful for the constructive comments and suggestions, which helped us improve the manuscript. Our detailed responses to each point are provided below.

- On page 3, line 36: the authors mentioned the importance of biotechnological application of T6SS. Authors could develop in more detail this issue.

Reply: We appreciate this suggestion. We have expanded this section in the revised manuscript by providing additional details and examples of potential biotechnological applications of the T6SS.

- Page 11, lines 26 – 32: In the same way, authors could provide more information on what is known considering the relationship between T6SS activity and resistance, since they provided only one reference on this subject.

Reply: We thank the reviewer for this comment. In this section, we cited only one reference linking T6SS to antimicrobial resistance because the focus of the text is on the regulatory aspects of T6SS expression, and this was the only relevant study in that context. However, a broader discussion on the correlation between T6SS presence and the antimicrobial resistance profiles of strains is provided in the section "T6SS genomic context and distribution." To clarify this for the reader, we have added a sentence on page 11 directing readers to that section for further discussion.

- Given the complexity of T6SS, it would be interesting to provide a table describing the main function of each T6SS component

Reply: We agree that clarifying the role of each T6SS component is important. This information was not initially included because it had already been presented in the only previous review specifically focused on *K. pneumoniae* T6SS (doi:10.2147/IDR.S426657). Nevertheless, to improve clarity for readers, we have now added Table I, which groups T6SS components according to the structural module to which they belong.

- Page 16, line 45: this sentence gives the idea that the T6SS would be one of the factors involved with the hypervirulent phenotype in *K. pneumoniae*. Be aware of that since, although T6SS contributes to *K. pneumoniae* pathogenesis, it is not a determinant of hypervirulence. It would be better to remove the reference 46 from this context.

Reply: We appreciate this observation. We have revised the sentence to avoid implying that T6SS is a determinant of hypervirulence.

Reviewer: 2

The manuscript by Lery and Batista is a comprehensive review of the current knowledge regarding the role, regulation, and effectors of the Klebsiella pneumoniae T6SS. Overall, the manuscript is well presented and represents an important contribution to the field. The abstract is adequate, and the figures and tables are valuable additions that greatly help the reader follow the information presented. I have some major points that should be addressed by the authors, as well as some minor revisions to the text, as outlined below.

Reply: We thank the reviewer for the careful evaluation of our manuscript and for the positive assessment, especially regarding its clarity and figures contribution. We are grateful for all the constructive comments which have helped us improve the manuscript. Our detailed responses to each point are provided below.

1) Some *K. pneumoniae* genomes may contain up to three T6SS clusters, including one complete cluster and two incomplete ones. This is an interesting observation that could benefit from further discussion in the manuscript. In particular, the authors do not provide information on the distribution and prevalence of these incomplete clusters. It would be helpful to clarify whether they are conserved across strains or present only in a subset of isolates. In addition, it would be important to specify which clusters are associated with the effectors described in the text. Finally, the authors may wish to comment on whether these incomplete clusters could represent a potential source of variation for the complete cluster.

Reply: We appreciate this comment. We appreciate this important comment. An additional paragraph further discussing these aspects has now been included in the section "T6SS genomic context and distribution."

2) Page 15, lines 39-55 (description of VgrG4 function): The text would benefit from revision to combine sentences that are currently too short. In addition, the mechanism of action of VgrG4 is not clearly explained in the manuscript. It would be helpful for the authors to clarify how VgrG4 induces mitochondrial dysfunction, activates immune receptors, and promotes actin remodeling in a ROS-dependent manner.

Reply: We have revised this paragraph to improve readability and provide a clearer mechanistic description of VgrG4 activity.

3) Page 18, lines 6-8: "Recently, Liu et al. described an hvKP MDR clinical isolate and attributed bacterial hypervirulence due to T6SS – as it lacked hypermucoviscosity": I did not understand the sentence and the correlation of T6SS and hypermucoviscosity. Also, authors mention an association of T6SS presence and biofilm formation but the information it not clearly presented.

Reply: We apologize for the lack of clarity in this sentence. Since T6SS is not recognized as a determinant of hypervirulence, we have revised this section to avoid this interpretation.

- Page 13, lines 3-13: The section describing the regulation of T6SS expression by Fur and IHF would benefit from additional clarification. In particular, it would be helpful to indicate whether these regulators act as positive or negative regulators and under which experimental conditions this regulation was observed. For example, does Fur repress T6SS expression under iron-replete conditions? Same for the text regarding BolA: please describe BolA and how does it regulate T6SS gene expression;

Reply: We appreciate this suggestion. We have clarified the direction of regulation (up- or down-regulation) mediated by these regulators and added additional details describing the role of BolA as a regulator of gene expression.

- Authors could also discuss whether some reported effects of lack of T6Ss activity on animal colonization by *K. pneumoniae* could be an indirect effect of reduced competitive fitness against the microbiota or a direct role of the T6SS in virulence (example: page 15, line).

Reply: We agree with the reviewer and have incorporated this discussion into the revised manuscript.

Minor text revisions:

- page 7, line 25 "Notably, in the *K. pneumoniae* 52.145 strain, K2 hypervirulent strain, one T6SS locus": revise to "Notably, in the *K. pneumoniae* 52.145 strain, a K2 hypervirulent strain, one T6SS locus"

Reply: The text has been revised accordingly.

-page 11, line 20: "CR-KP strains"- include the full description of the abbreviation

Reply: The missing information was included. The abbreviation has now been defined at its first occurrence. Thank you.

- Page 14, lines 23-28 (However, there are missing....): sentence needs revision.

Reply: The sentence has been revised.

- Page 15, line 3: revise to: "With respect to antibacterial activity, the Type VI lipase effector (Tle1) is the best described so far" or "most studied so far"

Reply: The sentence has been modified.

- page 15, lines 41,42: revise to remove the comma.("the authors selected 3 representative proteins containing domains not previously described as T6SS effectors,")

Reply: The sentence has been modified accordingly.

- Figure 3: my suggestion is to use a T6SS representation instead of a syringe

Reply: The syringe representation has been replaced with a T6SS schematic in Figure 3.

- Page 17, line 23: "Genomic diversity in *K. pneumoniae* is huge (39).": The information provided would benefit from greater specificity. For example, what is the overall size of the *K. pneumoniae* core genome?

Reply: We acknowledge this comment. Additional information regarding genomic diversity has been included in the section "T6SS genomic context and distribution."

- page 20, lines 18-22: "There is still a lack of systematic screenings efforts for molecules with such activity. Systematic screening efforts for T6SS inhibitors are still lacking.": revise to remove redundancy.

Reply: Thank you, the redundancy has been removed and the text was revised.

- page 20, line 33: *K. pneumoniae*

Reply: Modified.

# Reviewer report: REVIEWERS' COMMENTS

## REVIEWER #1

The authors addressed all comments and recommendations made by reviewers.

## REVIEWER #2

All the major points have been adequately addressed.
